# N7-methylguanosine-related lncRNAs: Predicting the prognosis and diagnosis of colorectal cancer in the cold and hot tumors

**DOI:** 10.3389/fgene.2022.952836

**Published:** 2022-07-22

**Authors:** Jing-Yu Wu, Qing-Yu Song, Chang-Zhi Huang, Yu Shao, Zhen-Ling Wang, Hong-Qiang Zhang, Zan Fu

**Affiliations:** ^1^ The General Surgery Laboratory, The First Affiliated Hospital of Nanjing Medical University, Nanjing, China; ^2^ The General Surgery Laboratory, The Second Affiliated Hospital of Nanjing Medical University, Nanjing, China

**Keywords:** colorectal cancer, N7-methylguanosine, lncRNAs, hot tumors, cold tumors, TCGA

## Abstract

**Background:** 7-Methylguanosine(m7G) contributes greatly to its pathogenesis and progression in colorectal cancer. We proposed building a prognostic model of m7G-related LncRNAs. Our prognostic model was used to identify differences between hot and cold tumors.

**Methods:** The study included 647 colorectal cancer patients (51 cancer-free patients and 647 cancer patients) from The Cancer Genome Atlas (TCGA). We identified m7G-related prognostic lncRNAs by employing the univariate Cox regression method. Assessments were conducted using univariate Cox regression, multivariate Cox regression, receiver operating characteristics (ROC), nomogram, calibration curves, and Kaplan-Meier analysis. All of these procedures were used with the aim of confirming the validity and stability of the model. Besides these two analyses, we also conducted half-maximal inhibitory concentration (IC50), immune analysis, principal component analysis (PCA), and gene set enrichment analysis (GSEA). The entire set of m7G-related (lncRNAs) with respect to cold and hot tumors has been divided into two clusters for further discussion of immunotherapy.

**Results:** The risk model was constructed with 17 m7G-related lncRNAs. A good correlation was found between the calibration plots and the prognosis prediction in the model. By assessing IC50 in a significant way across risk groups, systemic treatment can be guided. By using clusters, it may be possible to distinguish hot and cold tumors effectively and to aid in specific therapeutic interventions. Cluster 1 was identified as having the highest response to immunotherapy drugs and thus was identified as the hot tumor.

**Conclusion:** This study shows that 17 m7G-related lncRNA can be used in clinical settings to predict prognosis and use them to determine whether a tumor is cold or hot in colorectal cancer and improve the individualization of treatment.

## Introduction

Colorectal cancer (CRC), a significant public health danger worldwide, remains a significant burden (Siegel et al., 2021). The majority of CRC patients have been treated with surgery, radiation therapy, and chemotherapy (Brenner et al., 2014), which are insufficient to prevent colorectal cancer. There has been some advancement in immunotherapy for colorectal cancer (Ganesh et al., 2019), but resistance to immunotherapy still occurs at times (Galon and Bruni, 2019). As such, it is imperative to investigate how to enhance immunotherapy in CRC.

An extensive body of evidence suggests that RNA modification is crucial in regulating gene expression both during and after transcription (Boccaletto et al., 2018; Mathlin et al., 2020). The modification of RNA by all living organisms has been reported to be 163 in number (Courtney et al., 2019; Chen et al., 2021). It is an RNA modification of N7-methylguanosine (m7G), a methyl group is added to the seventh nucleotide of RNA. mRNA is more stable with m7G modification (Furuichi et al., 1977; Shimotohno et al., 1977). M7G is also thought to regulate cell differentiation (Lin et al., 2018). Aside from mRNA, the m7G modification can also be found on tRNAs, rRNAs, and miRNAs (Guy and Phizicky, 2014; Sloan et al., 2017; Pandolfini et al., 2019). The AGO gene and CYFIP1 has been reported and plays an important role in the development of colorectal cancer (Kim et al., 2010; Liu et al., 2018; Yan et al., 2019; Kennedy et al., 2020). The methylation complexes involved in m7G methylation include METTL1 and WDR4 (11).

It is estimated that approximately 16,000 of these genes were lncRNAs, accounting for about one quarter of the total number of human genes. In contrast to miRNA and snRNA, lncRNAs are longer and have a lower protein-coding potential (Rinn and Chang, 2012). In the past, researchers have suggested lncRNAs play a crucial role in regulating transcription and post-translational regulation, as well as chromatin modification (Wang et al., 2018). In studies published recently, aberrant lncRNA expression in tumors was examined as a diagnosis and prognosis marker (Poursheikhani et al., 2020). Moreover, Evidence is accumulating that LncRNAs contribute to tumor inflammation as well as assisting malignancies to evade immune destruction (Dragomir et al., 2020). The role of lncRNAs related to m7G as a potential therapeutic target has not been widely explored to date in the treatment of CRC. Consequently, we can gain greater insight into the roles of m7G and lncRNAs in immunotherapy by acquiring more lncRNA-related knowledge.

Immunotherapy will be more effective if cold tumors are transformed into hot tumors. This will lead to a breakthrough in immunotherapy, but at this stage, the mechanisms of RNA modification in CRC remain unclear. Nevertheless, there are still no simple and effective means for identifying cancerous tumors (Galon and Bruni, 2019). lncRNAs have been found to be highly accurate cancer markers, hence we decided that combining patients through m7G-related lncRNAs would improve clinical prediction and diagnosis (Meng et al., 2019; Yuan et al., 2020).

## Materials and methods

### Colorectal cancer data gathering

The Cancer Genome Atlas TCGA (http://portal.gdc.cancergov/) was used to study 647 colorectal cancer tissues and 51 control tissues. We excluded patients with colorectal cancer with missing overall survival (OS) and overall survival (OS) of less than 30 days from this analysis in order to avoid statistical bias. As a result of collecting pertinent clinical data, we randomly assigned 589 patients to two risk groups: train risk and test risk groups, respectively. Then we utilizing the R packages and caret Strawberry Perl. This proportion was 1:1.

### Genes selection and m7G-related lncRNAs

M7G gene set consists of 26 genes and was obtained from GSEA (http://www.gsea-msigdb.org/gsea/index.jsp). Furthermore, based on reports previously on m7G, 29 genes related to m7G have been collected (Supplementary Appendix SAT1). We first performed co-expression analysis of 29 M7g genes and LncRNAs in the colorectal cancer dataset from TCGA (Pearson correlation coefficients >0.4, and *p* < 0.001). Following this, we performed differential analysis of these LncRNAs (|Log_2_fold change (FC)| > 1, false discovery rate (FDR) < 0.05, and *p* < 0.05). We finally obtained the m7G-related lncRNAs to construct a prognostic model for colorectal cancer.

### The risk model establishment and validation

We applied univariate Cox proportional hazard regression analysis on the TCGA m7G-related lncRNAs, in order to identify lncRNAs related to survival (*p* < 0.05). Afterward, our final decision was made after applying 10-fold cross validation *via* Lasso regression with a 0.05 *p*-value for a total of 1,000 times, and then we determined our final result. The random stimulus was presented 1,000 times each to avoid overfitting. The model was developed following the presentation of the random stimulus. The curves for the receiver operating characteristic (ROC) of the model for 3, 1, and 2 years were computed. These are the data points that we used when calculating the risk score: ^k^

$$\mathrm{risk}\;\mathrm{score}=\sum_{k=1}^{n}\mathrm{coef}\left(\mathrm{IncRN}A^{k}\right)*\mathrm{expr}\left(\mathrm{IncRN}A^{k}\right)$$

the coef (lncRNAn) referred to a short form of the coefficient representing the correlation of lncRNAs with survival, and the expr (lncRNAn) applied to the expression level for lncRNAs. The median risk score was used as a criterion to divide the study participants into subgroups according to risk (Meng et al., 2019; Hong et al., 2020). To determine the prognosis, we utilized the Chi-square test to analyze the relationship between the model and clinical factors.

### Independence factors and receiver operating characteristics

The receiver operating characteristic (ROC) analysis was applied to measure the influence of multivariate (multi-Cox) or univariate (uni-Cox) regression on outcomes.

### Calibration and nomogram

To demonstrate whether our prediction was being matched with the actual result, we used the risk score, the age of the patient, and the stage of the tumor to construct a nomogram for 1-, 2-, and 3-years OS. To verify the consistency between the predictions and the results, we applied the Hosmer-Lemeshow test.

### Gene Set Enrichment Analyses

The gene set enrichment analysis is carried out on the m7G-related gene set (kegg.v7.4.symbols.gmt) with the help of the gene set enrichment analysis (GSEA) program (http://www.gsea-msigdb.org/gsea/login.jsp) software. This program is designed to analyze the statistically (*p* < 0.01) enriched pathways between the two categories. They are the high and low-risk categories.

### Investigating the tumor microenvironment and immune checkpoints

According to the results of the GSEA it was decided to assess immune-cell factors in patients at high risk based on the GSEA. TIMER 2.0 (http://timer.cistrome.org/) was used to compute the immune infiltration status of colorectal cancer patients from the TCGA using TIMER, CIBERSORT, XCELL, QUANTISEQ, MCPcounter, and EPIC algorithms. From the same web, we can obtain the infiltration profiles of all TCGA tumors. To compare the difference in levels of immune cells infiltrating into the body between the two categories, we used the Wilcoxon signed-rank test and the R packages limma and scales, along with the ggplot2 and ggtext packages. Bubble charts were used to present the findings (Hong et al., 2020). With the R package ggpubr, it was possible to compare immunological checkpoint activation between low- and high-risk categories.

### Models of clinical reactions to the treatment in an exploratory study

On the basis of the half-maximum inhibitory concentration (IC50) calculate by Genomics of Drug Sensitivity in Cancer (GDSC) (https://www.cancerrxgene.org) each colorectal cancer patient is then tested for the therapeutic response using the R package pRRophetic (Geeleher et al., 2014).

### Clusters analysis

Based on the prognostic lncRNA expression in colorectal cancer, we investigated molecular subgroups based on ConsensusClusterPlus (CC) R package (Wilkerson and Hayes, 2010). We used the Rtsne R package to carry out a principal component analysis (PCA), a T-distributed stochastic neighbor embedding (T-SNE), as well as a Kaplan-Meier survival analysis. Furthermore, the GSVA Base and R package pRRophetic were utilized to conduct immunity analysis and drug sensitivity comparisons.

## Results

### M7G-related LncRNAs in colorectal cancer from TCGA

**FIGURE 1:**
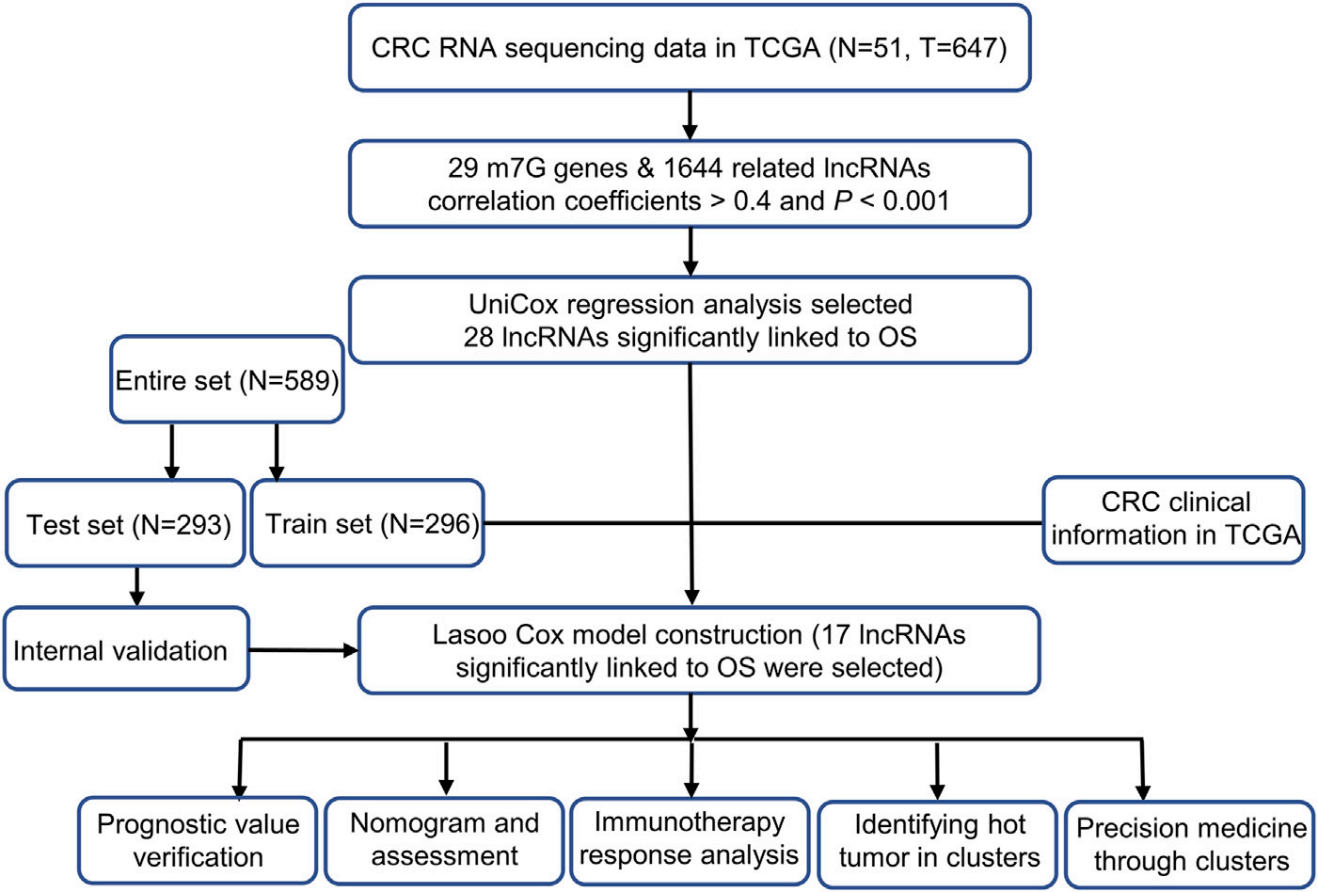
Study flow chart.

**FIGURE 2:**
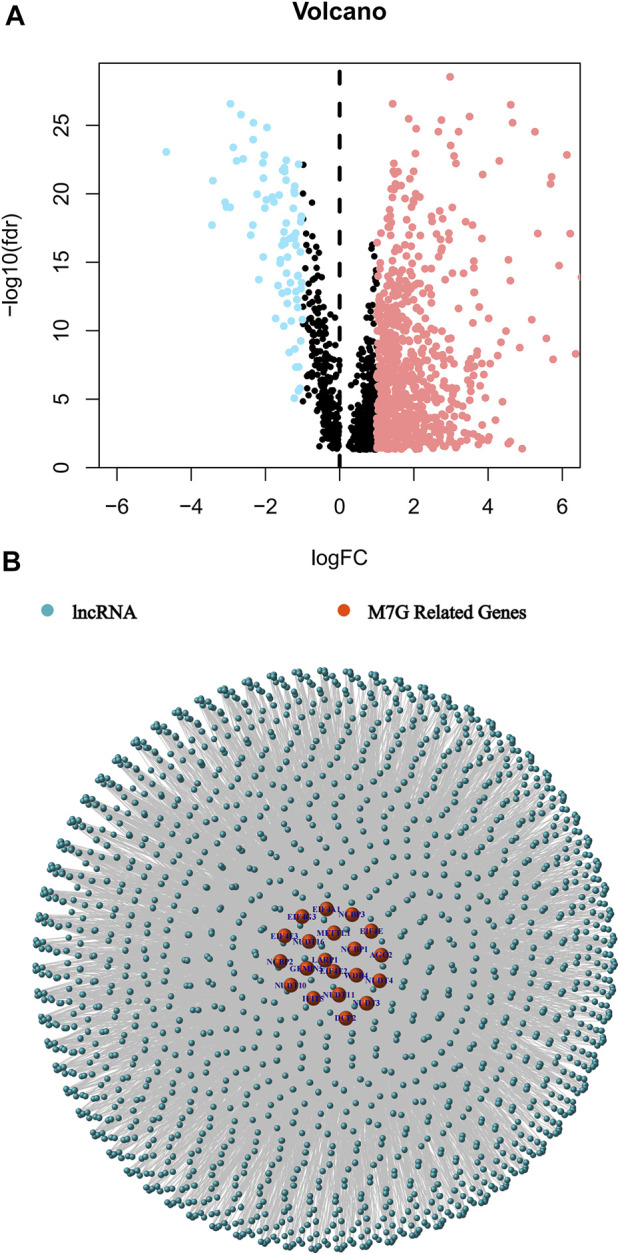
Identification of the m7G-related lncRNAs in patients with colorectal cancer. **(A)** The volcano plot of 827 differentially expressed m7G-related lncRNAs. **(B)** The network between m7G genes and lncRNAs (correlation coefficients >0.4 and *p* < 0.001).

The study’s flow was shown in Figure 1. We collected 51 colorectal cancer-free samples and 647 colorectal cancer samples from TCGA. Due to the expression of 29 m7G-related genes, as well as lncRNAs that were significantly different in expression (|Log_2_FC| > 1 and *p* < 0.05) between normal and tumor samples, we obtained 827 m7G-related lncRNAs (correlation coefficient >0.4 and *p* < 0.001) (Meng et al., 2019; Shen et al., 2020). 747 of them had an increase in expression, whereas the remaining 80 had a decrease in expression (Figure 2A). Supplementary Appendix SAD1 and Figure 2B demonstrate the network diagram and data between genes involved in m7G, such as EIF4E1B and METTL1.

### Model construction and validation

**FIGURE 3:**
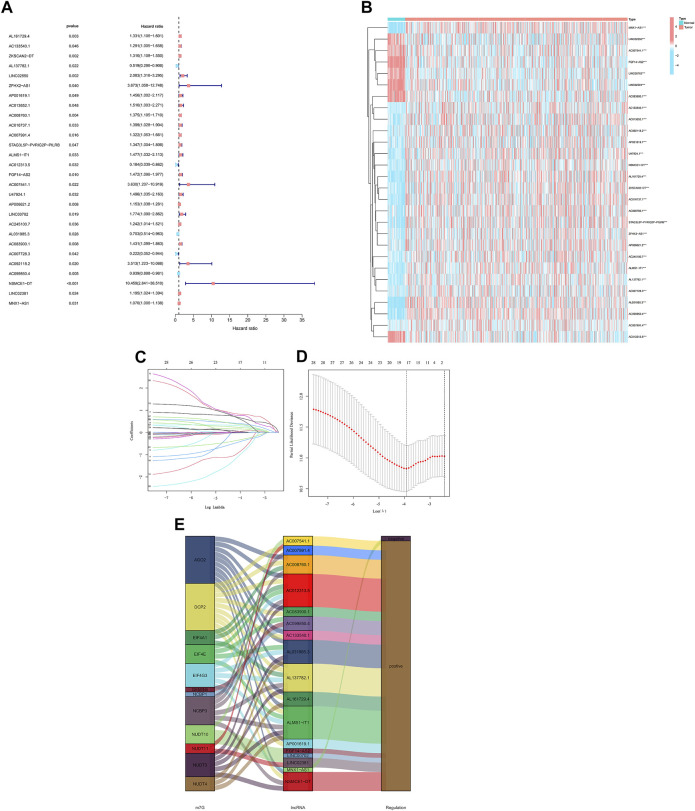
Analysis of the prognostic value of m7G-related lncRNAs in CRC. **(A)** Identification of prognostic lncRNAs using univariate Cox regression. **(B)** Prognostic lncRNA expression profiles of 28 genes. **(C)** Cross-validation of the LASSO model for variable selection in a 10-fold cross-validation procedure. **(D)** M7G-related lncRNAs LASSO coefficient profiles. **(E)** The Sankey diagram of lncRNAs related to m7G genes.

We identified 28 m7G-related lncRNAs that showed a statistically significant correlation with overall survival (OS) (all *p* < 0.05) using univariate Cox (uni-Cox) regression analysis and plotted the results (Figure 3A and Figure 3B). To prevent an overfitting of the prognostic signature, the Lasso regression technique was used. We identified 17 lncRNAs that were associated with m7G in colorectal cancer when the lowest deviation was achieved in Log(λ) for the first rank. (Figures 3C,D). In addition, the Sankey diagram revealed an upregulation of 16 lncRNAs (Figure 3E).

The formula we used for calculating risk score: riskscore = AL161729.4 × (0.0382) + AL137782.1 × (-0.3841) + ZFHX2-AS1 × (0.7618) + AP001619.1 × (0.6377) + AC008760.1 × (0.2205) + AC007991.4 × (0.2908) + ALMS1-IT1 × (0.5570) + AC012313.5 × (-0.7829) + FGF14-AS2 × (0.1546) + AP006621.2 × (0.0097) + AC245100.7 × (0.0587) + AL031985.3 × (-0.3110) + AC083900.1 × (0.3899) + AC007728.3×(-0.9178) + AC099850.4 × (-0.0122) + NSMCE1-DT × (0.8168) + LINC02381 × (0.3508).

**FIGURE 4:**
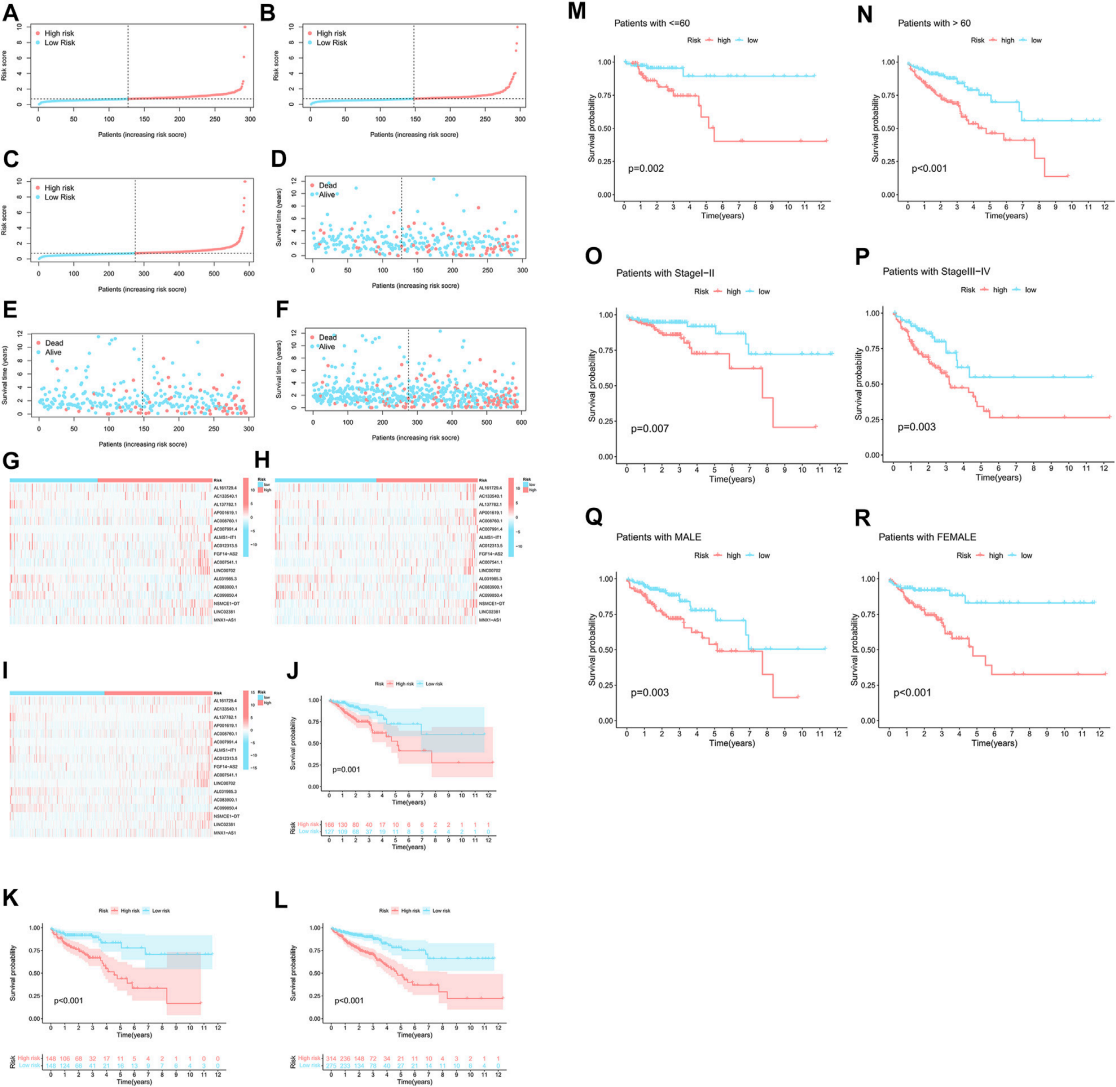
A high prognostic value is predicted for each of the 17 m7G-lncRNAs. **(A–C)** Display of the m7G-related lncRNAs based on tests, test results, and the complete set of m7G-related lncRNAs. **(D–F)** Comparison of the survival times and survival status in the train, test, and entire sets, respectively, for low- and high-risk groups. **(G–I)** The following is a heat map of the expression of 17 lncRNAs in the train set, test set, and entire sets. **(J–L)** For the train, test, and entire set sets of patients in low-risk and high-risk groups, respectively, Kaplan-Meier survival curves were generated for OS (survival probability). **(M–R)** Stratified by age, gender, or stages, Kaplan-Meier survival curves show OS (survival probability) for low-risk and high-risk groups in the entire sample.

As a comparative study of low- and high-risk populations. Utilizing the algorithm to compare the distribution of risk scores, survival statuses, and survival times as well as expression criteria for these lncRNAs in the train sets, test sets, and entire sets. According to all of these indicators, there was a poor prognosis for the high-risk populations (Figure 4A–L). As well as typical clinicopathological parameters (Figure 4M–R).

### Nomogram construction

**FIGURE 5:**
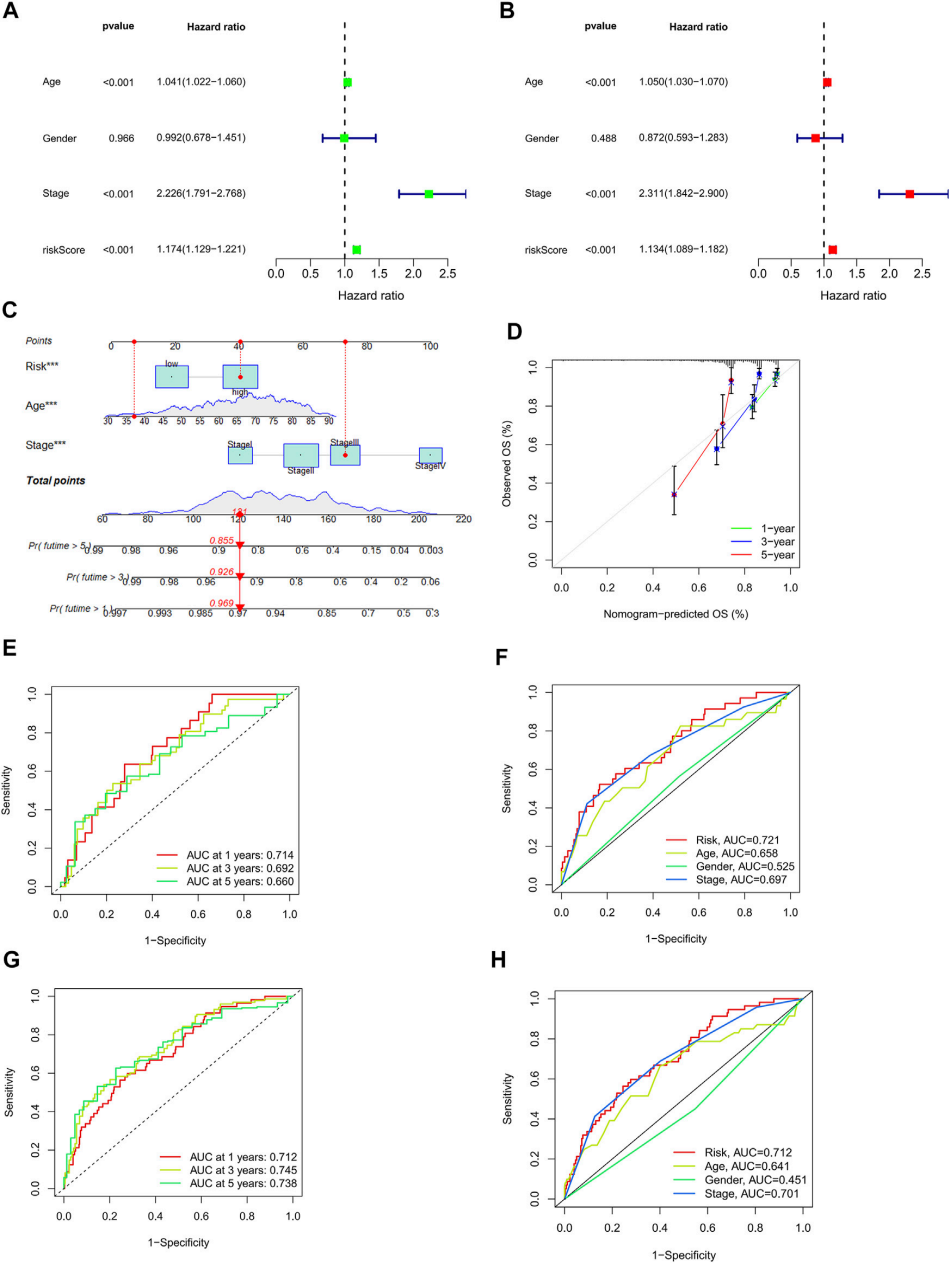
Assessment of the risk model and a detailed analysis of the nomogram. **(A,B)** Analysis of clinical factors and OS risk score using uni- and multi-cox models. **(C)** A nomogram incorporating the risk score, age, and stage of the tumor predicted the likelihood of survival at 1, 2, and 3-years intervals. **(D)** Calibration curves for the 1-, 2-, and 3-years OS. **(E–G)** The 1-, 2-, and 3-years ROC curves for the train, the test, and the entire set, respectively. **(H)** The 3-years ROC curves of risk score, and clinical characteristics.

In univariate Cox regression (HR = 1.174, 95% confidence interval (CI) = 1.129–1.221; *p* < 0.001) and in multivariate Cox regression (HR = 1.134, 95% confidence interval (CI) = 1.089–1.182; *p* < 0.001) (Figure 5A,B). Additionally, we detected two additional independent predictive variables: age (HR = 1.050 and 95% confidence interval (CI) = 1.030–1.070; *p* < 0.001) and stage (HR = 2.300 and 95% confidence interval (CI) = 1.842–2.900; *p* < 0.001). For CRC patients, our calculations were a nomogram based on three independent prognostic factors that can be used to predict their 1-, 2-, and 3-years survival: risk score (*p* < 0.001), age (*p* < 0.001), and stage (*p* < 0.001) in multi-Cox (Figure 5C). Furthermore, the first, second, and third-year calibration plots showed that the nomogram closely approximated predicting 1-, 2-, and 3-years OS. (Figure 5D).

### The risk model evaluation

The prognostic models were carried out with time-dependent receiver operating characteristics (ROC). Furthermore, the results are presented using the area under the ROC curve (AUC). The results of the ROC analysis were also illustrated with an area under the ROC curve (AUC). The 1-, 2-, and 3-years AUC of the train set were 0.721, 0.804, and 0.805, of the test set were 0.714, 0.692, and 0.660, and of the entire set were 0.712, 0.745, and 0.738, respectively. (Figures 5E–G). In an analysis of the 3-years ROC curve, the risk model, clinical factors, and nomogram total score, the risk model (0.712) demonstrated the highest predictive capability of the data (Figure 5H).

### GSEA

**FIGURE 6:**
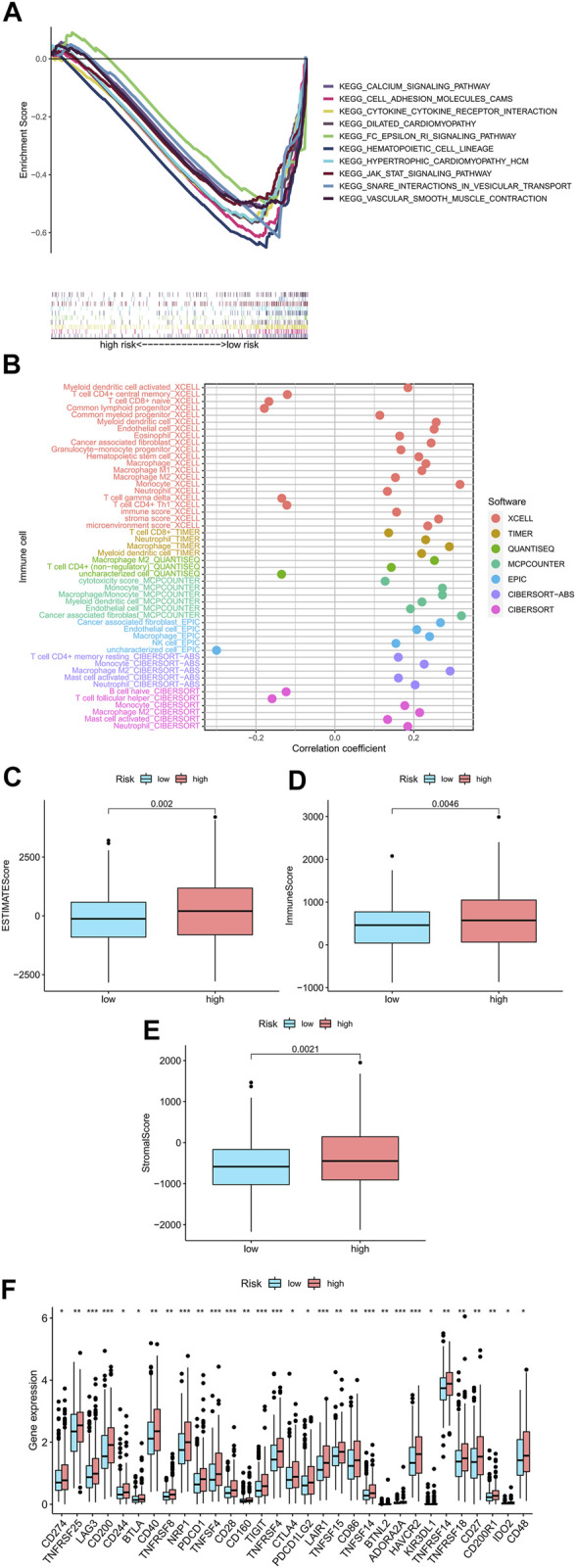
Research on tumor immune factors and immunotherapy. **(A)** A significant enrichment of GSEA was discovered in the high-risk group for the pathways that were identified in the top ten pathways. **(B)** Immune cell bubbles in risk groups. **(C–E)** Comparing immunity scores in low- and high-risk groups. **(F)** Different expressions of 15 checkpoints in risk groups.

Using GSEA software, in order to determine whether there were differences in biological functions in the high-risk patients, we performed an analysis on the KEGG pathway for the high-risk patients (Supplementary Fig 3). Among high-risk patients, in the top 10 pathway list, eight of them had a significant correlation with tumor invasion, whereas the others, such as “cytokine receptor interaction,” had a significant association with immunity (all *p* < 0.01; |NES| > 1.5) (Figure 6A) (Shen et al., 2020). Due to this, we attempted to analyze the model based on immunity.

### Studying immunity factors and clinical strategies for high-risk groups

On several platforms, such as T cell CD4^+^ memory, Macrophage M1 at XCELL, T cell CD8^+^, Macrophage at TIMER, Macrophage M1 at QUANTISEQ, and cancer-associated fibroblast at MCPcounter and EPIC, showed strong associations with high-risk patients. (all *p* < 0.05) (Figure 6B) (Supplementary Appendix SAD2). The majority of immune cells were also more activated among high-risk patients. According to these findings, the high-risk patients displayed a higher level of immunological infiltration (all *p* < 0.05) (Figure 6C–E). Almost all immune checkpoints were activated in the high-risk group (Figure 6F), which means that colorectal cancer patients can be treated with immune checkpoint inhibitors based on the risk model (Johdi and Sukor, 2020). Additionally, we discovered that 16 chemical or targeted medicines (all *p* < 0.001) (Supplementary Fig 1), including Shikonin.

### Identifying cold and hot tumors and medicine

**FIGURE 7:**
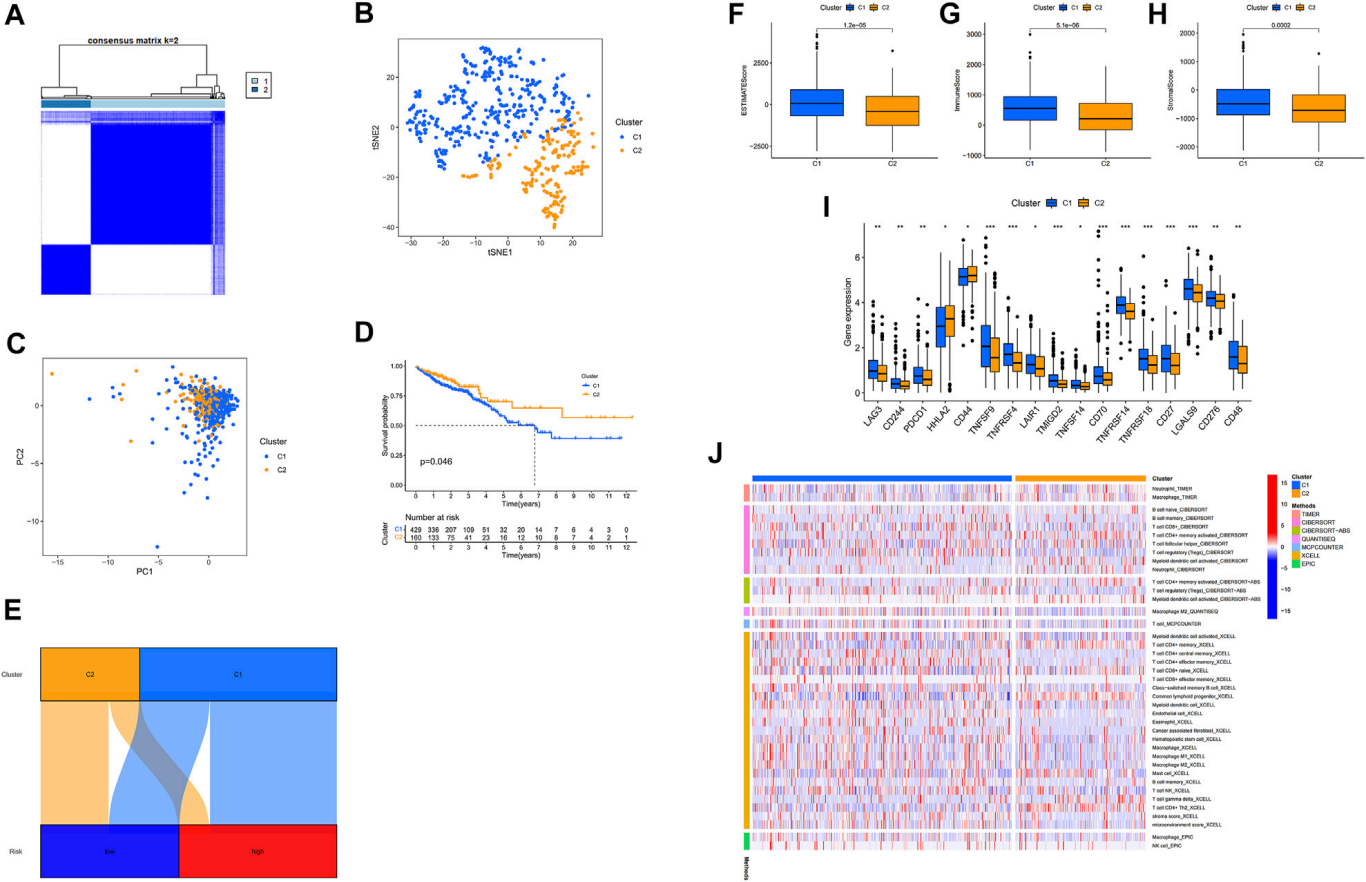
Identifying cold and hot tumors and predicting their response to immunotherapy. **(A)** The Consensus Cluster Plus algorithm divides patients into two clusters. **(B)** Two clusters of t-SNE. **(C)** PCA of clusters. **(D)** Based on Kaplan-Meier survival curves, OS survival curves for clusters of patients. **(E)** Cluster analysis of risk groups. **(F–H)** Scores in immune-related categories between clusters 1 and 2 are compared. **(I)** The difference between 32 checkpoint expressions in clusters. **(J)** Clusters of immune cells on a heat map.

Previously published reporters have demonstrated that there are different types and subtypes of clusters that are usually associated with different immune microenvironments, resulting in various immunotherapeutic effects (DeBerardinis, 2020; Shen et al., 2020). Two clusters of patients were derived based on 17 m7G-related lncRNAs using the ConsensusClusterPlus (CC) R package (Figure 7A) and Supplementary Fig 4 (27). As a result of the T-distributed stochastic neighbor embedding (t-SNE), we have been able to discern two clusters (Figure 7B). Additionally, we carried out Principal Component Analysis (PCA) to check that the PCA values for clusters were different (Figure 7C). Further, A better OS was identified in cluster 2 (*p* = 0.046) in the Kaplan-Meier analysis (Figure 7D). Also, a chart was made to verify its relationship with risk. Cluster 1 were significantly associated with high risk, whereas cluster 2 were significantly associated with low risk (*p* < 0.001) (Figure 7E). These results from Cluster 1 below can provide important insights into how to treat patients in high-risk groups. Cluster 1 was significantly infiltrated by immune cells, as determined by the analysis of the different platforms (Figure 7J) (Supplementary Appendix SAD3). There was a significant association between cluster 1 and a high ESTIMATE score and immune score over cluster 2, indicating that this is a distinct TME (Figures 7F–H). In cluster 1, almost all immune checkpoints, such as CD27, LAG3, and TNFRSF18, displayed higher activity (Figure 7I). The CD8^+^ T cells, the inflammation-promoting function, the high immunity score, the activation of CD27, LAG3, and TNFRSF18, played a critical role in the hot tumour. Therefore, clusters 1 and clusters 2 may be considered hot and cold tumors, respectively (Galon and Bruni, 2019; Zheng et al., 2021). Different immunotherapeutic responses may result from it (Zeng et al., 2019; DeBerardinis, 2020). Cluster 1 was considered more susceptible to immunotherapy in view of the concept of cold and hot tumors. Our findings showed that 17 compounds that were effective as systemic treatments for CRC that were effective as individualized treatments for CRC. (Supplementary Fig 2) (Boulos et al., 2019; Li et al., 2020). Based on clusters derived from these lncRNAs, we may be able to investigate immunotherapy responses and enhance the efficiency of specific therapy in patients with CRC.

## Discussion

Immunotherapy may be beneficial as a result of TME immunosuppressive properties, a situation which is frequently associated with treatment failure. However, there is no panacea for all diseases. Some patients have had poor results following immunotherapy (Tang et al., 2020). In order to improve the effectiveness of immunotherapy, we developed the concept of cold and hot tumors to distinguish between cancer and immune-based tumor classification. Infiltration rates and immune scores of tumors are generally considered hot and cold, respectively, depending on their infiltration rate and immune score. Another characteristic of a hot tumor is the higher activity of checkpoint proteins as well as its more aggressive nature, such as PDCD1, which is another sign of a hot tumor. Patients who are suffering from a hot tumor can be treated with immunotherapies targeting CD8^+^ T cells, microbiome modulation, or other immunotherapeutic therapies. However, because cold tumors do not release any immunity through low-level T cells, they are often very difficult to battle. Enhanced immune therapy *via* the PD-1/PD-L1 pathway can kill cancer cells and break tolerance, which in turn boosts anti-tumor immunity through the release of CD8^+^ T cells. Rather than merely administering other treatments to a cold tumor, it is prudent to turn it into a hot tumor.

According to our study, we synthesized 17 m7G-related lncRNAs and attempted to determine whether cold or hot tumors. After re-grouping patients, some analyses were conducted, based on the model, such as GSEA analysis. However, a risk group-based analysis could not determine the hot tumor, suggesting that prognosis and treatment can be predicted using risk groups. Recent studies have demonstrated that tumor immunity suppression and microenvironments are associated with molecular subtypes, also called clusters (Ajani et al., 2017). Accordingly, each subtype has a different immune system and TME score, which results in different prognoses and responses to immunotherapy (DeBerardinis, 2020). Using these lncRNAs, we divided patients into two clusters based on their expression (Wilkerson and Hayes, 2010). We found that the immune microenvironments of the two groups differed. The immune microenvironment of cluster 2 was immunosuppressive. It is possible to classify the hot tumors in cluster 1 as having more PD-L1 (4, 31). Cluster 1 was also more sensitive to immunotherapeutic drugs than cluster 1. In addition to predicting prognosis, m7G-related lncRNAs can also applied to guide individual treatment. In contrast to a mass cytometry or other means of a tumor biopsy, the lncRNAs can be used as liquid biopsies for detecting different tumor types (Duan et al., 2020).

A number of cancer researchers are exploring the possibility of using long noncoding RNAs as prognostic biomarkers. Among the 17 lncRNAs related to m7G identified in our study, only AL137782.1, AC012313.5, AL031985.3, AC007728.3 and AC099850.4 are protective factors, but the other lncRNAs were risk factors. Consistent with our results, Previous reports have shown that AC133540.1, AL137782.1, AP001619.1, AC013652.1, AC008760.1, ALMS1-IT1, AC012313.5, FGF14-AS2, AP006621.2, LINC00702, LINC02550 and AC083900.1 are associated with poor prognosis in CRC patients (Huang and Pan, 2019; Hou et al., 2020; Yu et al., 2020; Xu et al., 2021a; Li et al., 2021; Li et al., 2022), whereas AC007991.4, LINC02381, AL031985.3 and AC099850.4 were reported in the other cancer patients with poor prognosis (Jafarzadeh and Soltani, 2020; Zhang et al., 2020; Zhao et al., 2020; Wu et al., 2021). The other lncRNAs were initially discovered. We may be able to develop a better understanding of how m7G-related lncRNAs contribute to CRC *via* novel biochemical mechanisms, resulting in new therapeutic advances.

Even after utilizing many methods to asset our model, there were still some shortcomings and deficiencies. A retrospective study is prone to biases inherent in the paradigm for which it was undertaken, since it was carried out (Jiang et al., 2016). We were unable to compare IC50s for corresponding checkpoint inhibitors, for instance PD-1 inhibitors, because insufficient data on GDSC existed, despite significant differences between risk groups and clusters in checkpoint activation. Verifying the prognoses was carried out internally by examining the tests and entire sets of data in the model, however external validation proved to be problematic. The bubble and heat map of immune cells showed external validation from multiple platforms (Hong et al., 2020; Xu et al., 2021b). Our plan is to gather more clinical datasets in order to better establish their usefulness in the research field of m7G-related lncRNAs. Additionally, we verified the relationship between the m7G gene and the related lncRNAs in the m7Ghub (Song et al., 2020) and came up with inconsistent results. There was a lack of molecular mechanism research in our study. Identifying which m7G-related lncRNAs could regulate colorectal cancer survival is just the beginning. We will explore the specific mechanism of the screened lncRNAs that affected CRC progression in our next work.

Our findings indicate that m7G-related lncRNAs are strongly associated with colorectal cancer, and that our model of 17 m7G-associated lncRNA constructs has potential as an independent prognostic molecular signature to distinguish between hot and cold tumors in colorectal cancer. Besides making progress in immunotherapy, it will also contribute to cancer research.

## Conclusion

We would make a huge leap in improving patients’ prognoses and making great progress in individualized treatment if we could identify cold and hot tumors and tailor a therapeutic approach based on m7G-related long noncoding RNAs. A central concept of m7G and lncRNA development allowing immunotherapy to expand has been demonstrated in that they have the capability of overriding the failures associated with systemic treatments. In order to fully investigate and validate the relationships between m7G, lncRNA, immunity, and CRC, the mechanisms underlying these relationships need to be fully described and validated.

## Data Availability

The datasets presented in this study can be found in online repositories. The names of the repository/repositories and accession number(s) can be found in the article/Supplementary Material.

## Abbreviations

CRC, colorectal cancer; m7G, 7-Methylguanosine; TME, the tumor microenvironment; lncRNA, long noncoding RNA; GSEA, Gene Set Enrichment Analyses.
