# Supplementary figures and images for: N7-methylguanosine-related lncRNAs: Predicting the prognosis and diagnosis of colorectal cancer in the cold and hot tumors
Source: Front Genet. 2022 Jul 22;13:952836. doi: 10.3389/fgene.2022.952836 (PMC9352958; doi:10.3389/fgene.2022.952836)

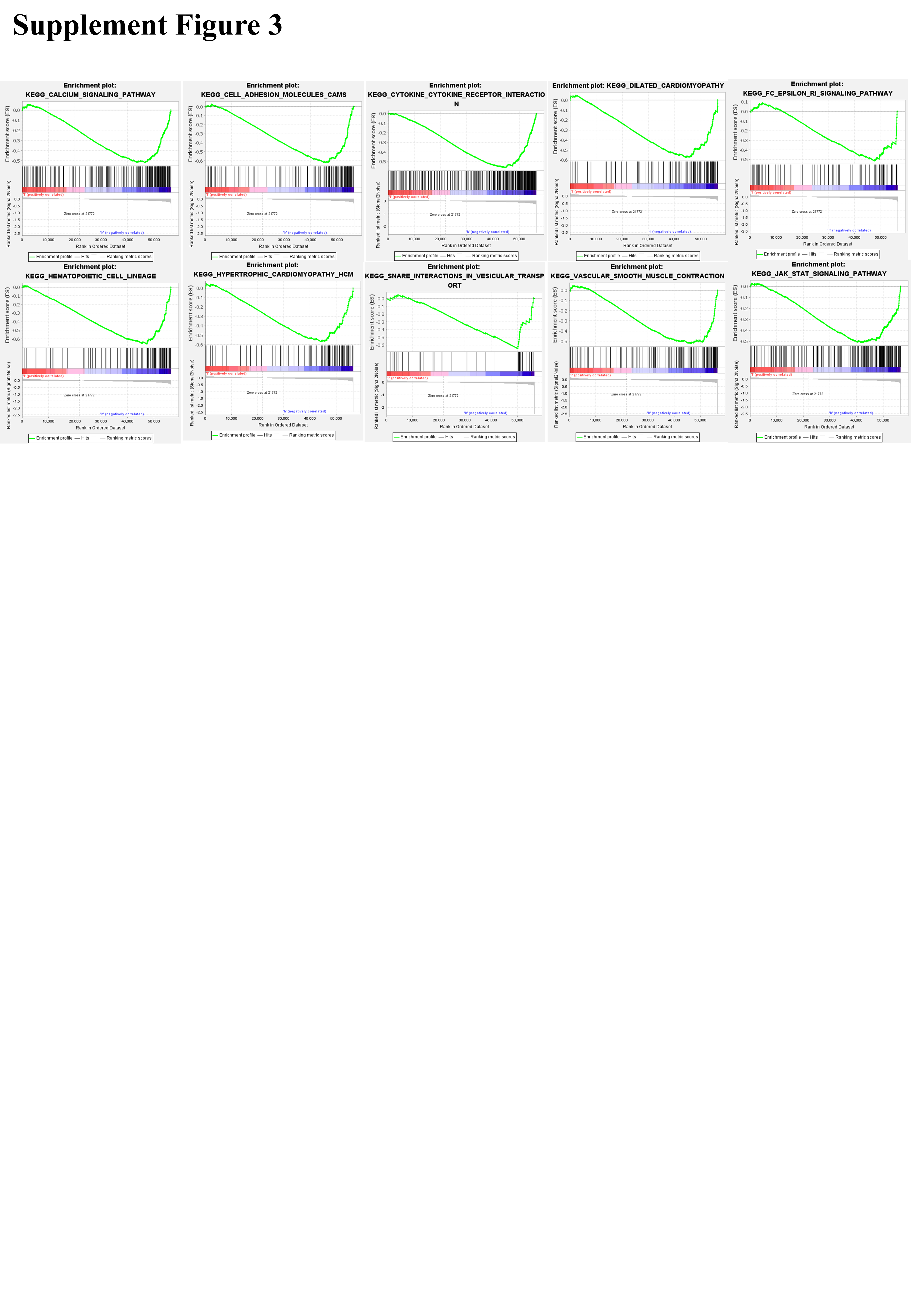

Supplement: Supplementary file 1 [file Image3.TIF]

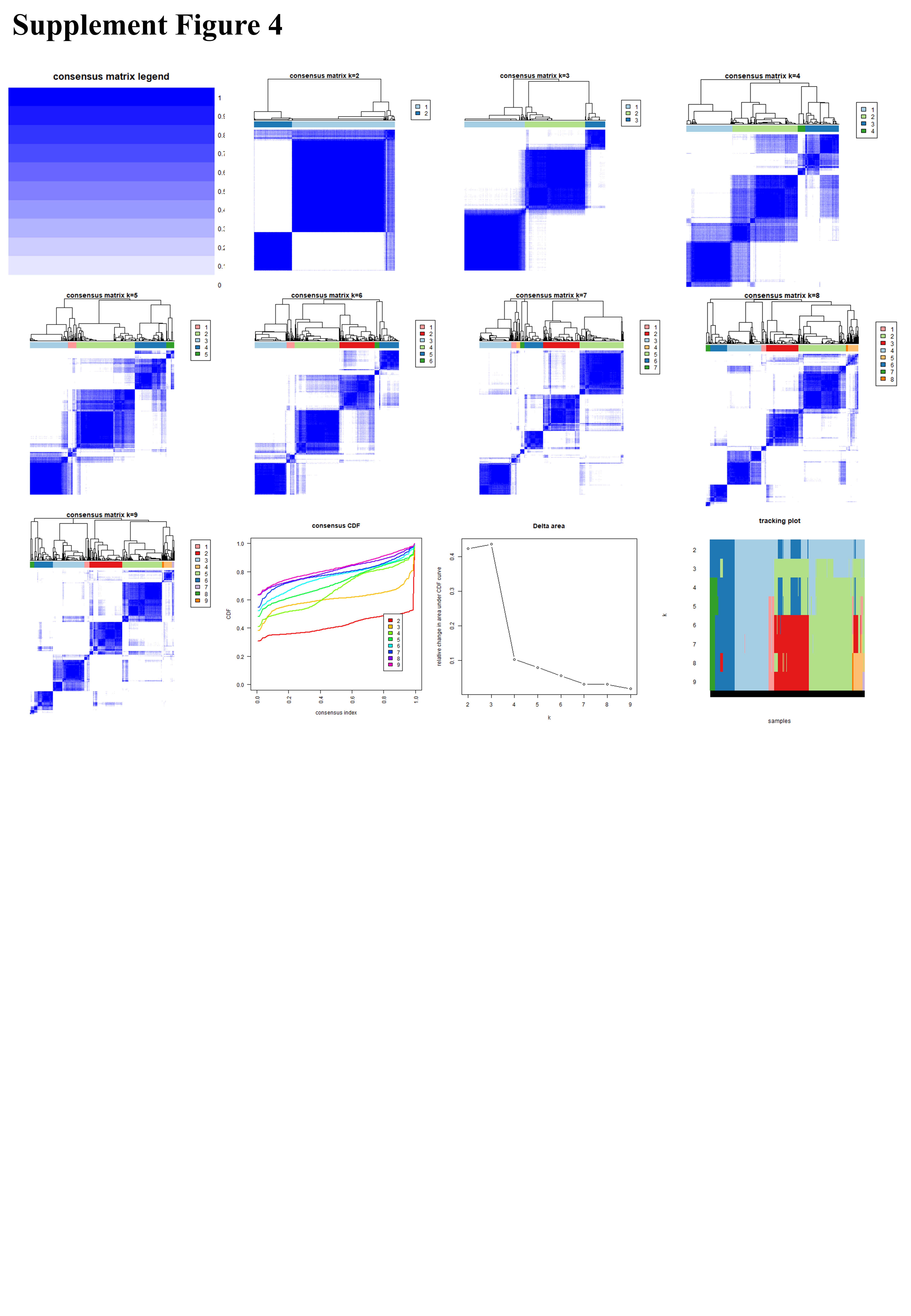

Supplement: Supplementary file 2 [file Image4.TIF]

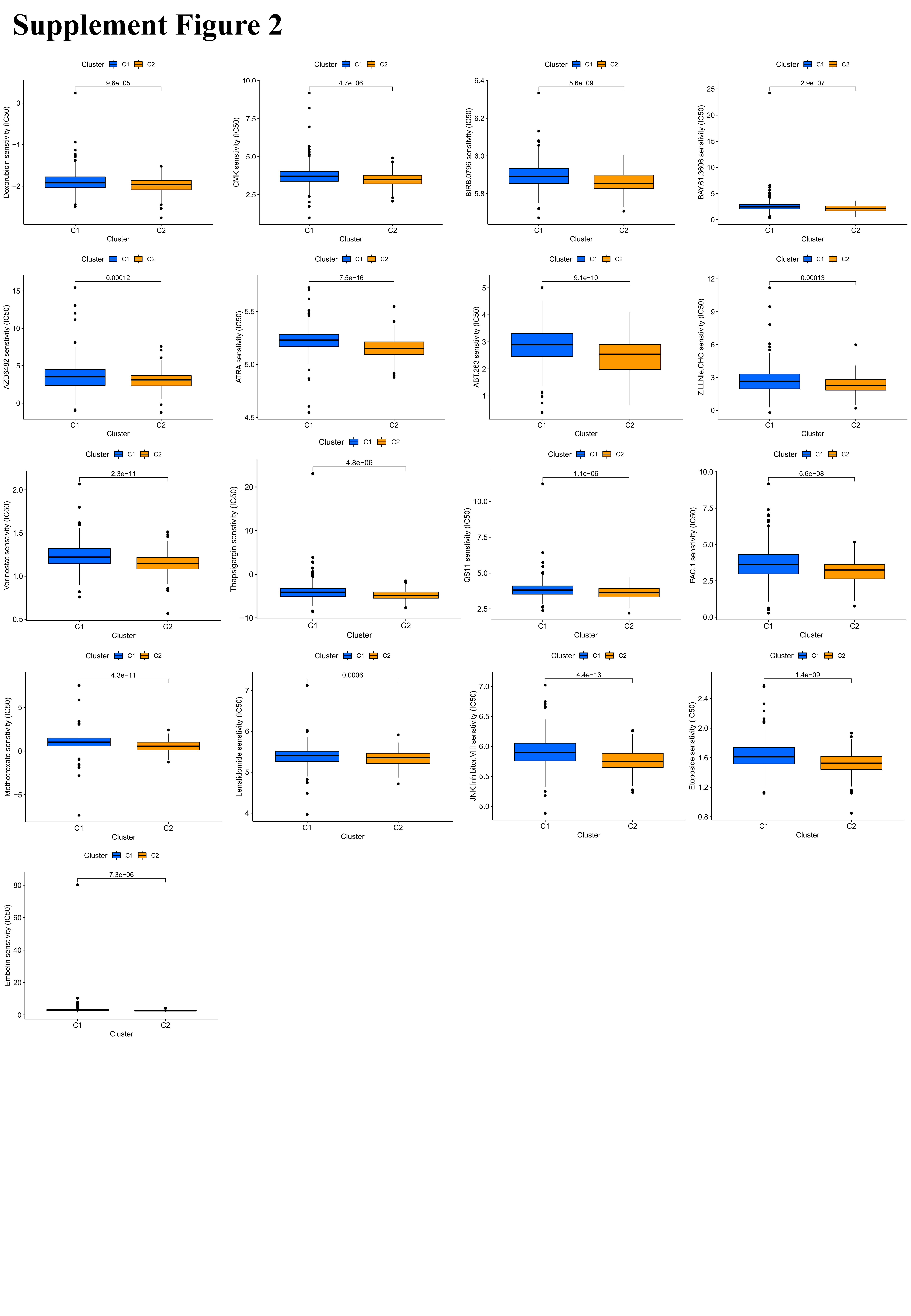

Supplement: Supplementary file 3 [file Image2.TIF]

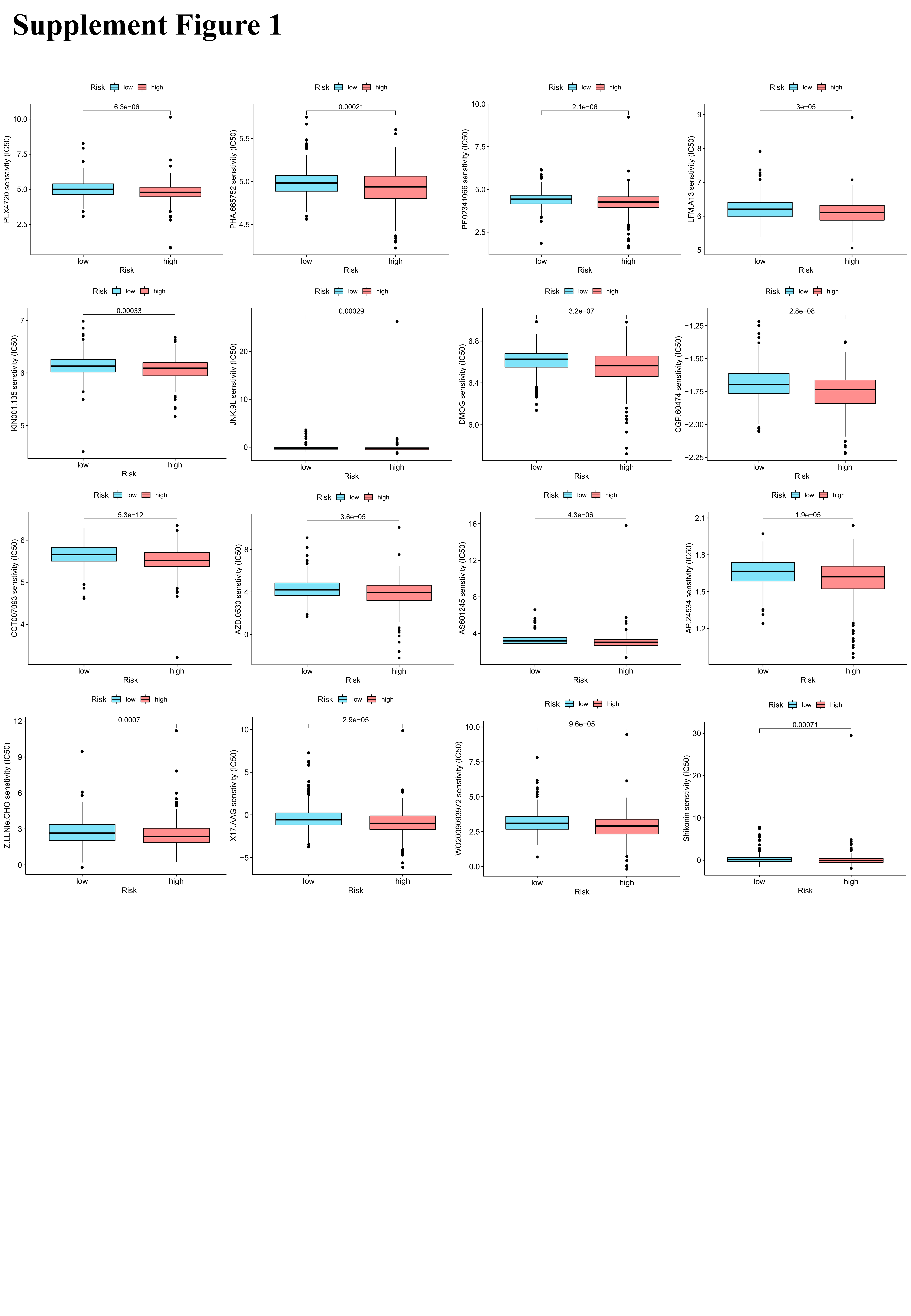

Supplement: Supplementary file 4 [file Image1.TIF]
