# Supplementary material for: N7-methylguanosine-related lncRNAs: Predicting the prognosis and diagnosis of colorectal cancer in the cold and hot tumors
Source: Front Genet. 2022 Jul 22;13:952836. doi: 10.3389/fgene.2022.952836 (PMC9352958; doi:10.3389/fgene.2022.952836)
Supplement: Supplementary file 5 [file Table6.DOCX]

**Supplementary Figure1:** IC50 predictions have been made for 16 compounds for risk groups.

**Supplementary Figure2:** IC50 predictions for 16 chemical or targeted drugs in cluster groups.

**Supplementary Figure3:** The GSEA of the high-risk group.

**Supplementary Figure4:** There are three heat maps, a cumulative distribution function plot, and the consensus CDF plots of the consensus clustering matrix.
